# The influence of spatial resolution on the spectral quality and quantification accuracy of whole‐brain MRSI at 1.5T, 3T, 7T, and 9.4T

**DOI:** 10.1002/mrm.27746

**Published:** 2019-04-01

**Authors:** Stanislav Motyka, Philipp Moser, Lukas Hingerl, Gilbert Hangel, Eva Heckova, Bernhard Strasser, Korbinian Eckstein, Simon Daniel Robinson, Benedikt A. Poser, Stephan Gruber, Siegfried Trattnig, Wolfgang Bogner

**Affiliations:** ^1^ High Field MR Centre, Department of Biomedical Imaging and Image‐guided Therapy Medical University of Vienna Vienna Austria; ^2^ Department of Radiology, Martinos Center for Biomedical Imaging Massachusetts General Hospital, Harvard Medical School Boston Massachusetts; ^3^ Department of Cognitive Neuroscience, Faculty of Psychology and Neuroscience Maastricht University Maastricht Netherlands; ^4^ Maastricht Brain Imaging Centre, Faculty of Psychology and Neuroscience Maastricht University Maastricht Netherlands; ^5^ Christian Doppler Laboratory for Clinical Molecular MR Imaging Medical University of Vienna Vienna Austria

**Keywords:** *B*_0_ field dependency, *B*_0_ inhomogeneities, MR spectroscopic imaging, signal‐to‐noise, spectral resolution, voxel size

## Abstract

**Purpose:**

Inhomogeneities in the static magnetic field (*B*_0_) deteriorate MRSI data quality by lowering the spectral resolution and SNR. MRSI with low spatial resolution is also prone to lipid bleeding. These problems are increasingly problematic at ultra‐high fields. An approach to tackling these challenges independent of *B*_0_‐shim hardware is to increase the spatial resolution. Therefore, we investigated the effect of improved spatial resolution on spectral quality and quantification at 4 field strengths.

**Methods:**

Whole‐brain MRSI data was simulated for 3 spatial resolutions and 4 *B*_0_s based on experimentally acquired MRI data and simulated free induction decay signals of metabolites and lipids. To compare the spectral quality and quantification, we derived SNR normalized to the voxel size (nSNR), linewidth and metabolite concentration ratios, their Cramer‐Rao‐lower‐bounds (CRLBs), and the absolute percentage error (APE) of estimated concentrations compared to the gold standard for the whole‐brain and 8 brain regions.

**Results:**

At 7T, we found up to a 3.4‐fold improved nSNR (in the frontal lobe) and a 2.8‐fold reduced linewidth (in the temporal lobe) for 1 cm^3^ versus 0.25 cm^3^ resolution. This effect was much more pronounced at higher and less homogenous *B*_0_ (1.6‐fold improved nSNR and 1.8‐fold improved linewidth in the parietal lobe at 3T). This had direct implications for quantification: the volume of reliably quantified spectra increased with resolution by 1.2‐fold and 1.5‐fold (when thresholded by CRLBs or APE, respectively).

**Conclusion:**

MRSI data quality benefits from increased spatial resolution particularly at higher *B*_0_, and leads to more reliable metabolite quantification. In conjunction with the development of better *B*_0_ shimming hardware, this will enable robust whole‐brain MRSI at ultra‐high field.

## 1 INTRODUCTION

MRSI is a non‐invasive method to map concentrations of various biochemical compounds in tissue. Since its introduction, MRSI has evolved into a unique tool that can add valuable information about pathology in many brain disorders.^1^, ^2^, ^3^, ^4^, ^5^ In some cases MRSI can even unravel biochemical changes, where conventional MRI appears insufficient.^6^

The quality of MRSI data depends strongly on the homogeneity of the main magnetic (*B*_0_) field, particularly that within each voxel.^7^ *B*_0_ inhomogeneity (Δ*B*_0_) increases spectral linewidths, decreases SNR, and leads to poor performance of frequency‐selective (suppression) pulses. Δ*B*_0_ are more severe at higher *B*_0_, and therefore diminish the expected improvements in SNR and spectral resolution when moving to a higher *B*_0_.^8^

To address these problems, Δ*B*_0_ are typically mitigated by superposition of additional magnetic *B*_0_ fields in the form of spherical harmonics^9^ and more recently by matrix *B*_0_ shims.^10^ One approach—independent of *B*_0_ shimming hardware—is to increase the spatial resolution, which reduces intra‐voxel Δ*B*_0_, but the ability to do so is limited by the available SNR per voxel and the maximum acceptable acquisition time.^11^, ^12^, ^13^ The associated spectral resolution improvements have been experimentally investigated in previous studies at ≤3T, but the effects are expected to be even more pronounced beyond 3T.

With the recent advent of whole‐brain MRSI at 7T and 9.4T,^14^, ^15^ and results that highlight the impact of high‐resolution MRSI in terms of pathological sensitivity^16^, ^17^ at 7T as well as below 7T,^18^, ^19^, ^20^, ^21^ it is critical to gain a better understanding of these mechanisms at ultra‐high *B*_0_ fields to be able to appropriately optimize whole‐brain MRSI protocols. This should be done in a manner that is insensitive to unrelated variations between measurements and where a “gold standard” is available for comparison.

In this study, we created whole‐brain MRSI simulation models based on MRI data acquired at 1.5T, 3T, 7T, and 9.4T. These simulation models allowed us to investigate not only how the spectral quality of the MRSI data but also the quantification accuracy, change with spatial resolution, and *B*_0_ field.

## 2 THEORY

The decrease of spectral quality in MRS/MRSI because of either increased Δ*B*_0_ or *T*_2_ shortening is indirectly proportional to the $T_{2}^{*}$ relaxation constant. The theory underlying this effect is described in the following sections.

### 2.1 *T*_2_ and *T*_2_^*^ relaxation

The key contributor to spectral quality improvements or degradation is $T_{2}^{*}$ relaxation. The measured FID signal at location r in the presence of Δ*B*_0_ is described by Equation 1, where the $T_{2}^{*}$ relaxation constant represents the total signal loss because of dephasing over time

(1) $$M_{\mathrm{xy}}\left(\text{r},t\right)=M_{\mathrm{xy}}\left(\text{r},0\right)e^{-t/T_{2}^{*}}.$$

$T_{2}^{*}$ correlates inversely with the broadening of spectral resonances, which determines the associated SNR and spectral resolution loss. The $T_{2}^{*}$ constant can be separated into 2 main contributions as shown in Equation 2: the apparent $T_{2,\text{apparent}}$ constant and the $T_{2,\text{macro}}$ constant. The $T_{2,\text{macro}}$ constant represents the macroscopic Δ*B*_0_ inside a voxel volume and therefore can be influenced by changing the spatial resolution in MRSI. Equation 1 can be rewritten as Equation 3, where ${\Delta B}_{0}\left(\text{r}\right)$ represents spatially dependent Δ*B*_0_.

(2) $$\frac{1}{T_{2}^{*}}=\frac{1}{T_{2,\text{apparent}}}+\frac{1}{T_{2,\text{macro}}},$$

(3) $$M_{\mathrm{xy}}\left(\text{r},t\right)=M_{\mathrm{xy}}\left(\text{r},0\right)e^{-t/T_{2,apparent}}\int_{r}e^{-i\gamma \Delta B_{0}\left(\text{r}\right)t}dr.$$

The $T_{2,\text{apparent}}$ constant is described by Equation 4. The intrinsic $T_{2,\text{intrinsic}}$ represents the homonuclear dipole–dipole interaction between protons, the hyperfine contact interaction with the paramagnetic center, and cross‐relaxation. The $T_{2,\text{Diffusion}}$ and the $T_{2,\text{Exchange}}$ describe the contribution of dynamic dephasing, where net magnetization is reduced by diffusion and exchange between regions with different magnetic field strengths.^22^ Neither can be experimentally altered. The contribution of dipole–dipole interactions associated with the $T_{2,\text{intrinsic}}$ constant is *B*_0_‐independent, but the remaining effects lead to an overall decrease of $T_{2,\text{apparent}}$ with increasing *B*_0_.^23^

(4) $$\frac{1}{T_{2,\text{apparent}}}=\frac{1}{T_{2,\text{intrinsic}}}+\frac{1}{T_{2,\text{Diffusion}}}+\frac{1}{T_{2,\text{Exchange}}}.$$

### 2.2 Spectral quality parameters

High‐quality spectra are generally characterized by metabolite signals, which are large compared to the noise level and well‐separated. These spectral quality features can be quantitatively described by the SNR (i.e., signal amplitude divided by the SD of noise) and the full‐with‐at‐half‐maximum (FWHM) of resonance peaks as a measure of linewidth and/or spectral resolution. The quality of the spectra depends strongly on various acquisition parameters.

The signal amplitude is theoretically expected to increase linearly with *B*_0_ because of the Zeeman effect. However, to cover all the important metabolite signals, it is necessary to increase the spectra bandwidth (in Hz) linearly with increasing *B*_0_ field. To achieve a linear increase of SNR, with the assumption of a fixed ADC length, the signal FID has to be multiplied by *B*_0_ and the SD of the noise, σ, modified according to Equation 5

(5) $$\sigma_{\mathrm{lowBW}}=\sigma_{\mathrm{highBW}}\sqrt{\frac{\mathrm{lowBW}}{\mathrm{highBW}}}.$$

The spatial resolution defines the volume of the signal origin. With decreasing volume, the signal amplitude also decreases. For MRSI with spatial (phase) encoding in 3 dimensions, the SNR per volume is described by Equation 6.

(6) $$SNR/voxel\propto \Delta x\Delta y\Delta z\sqrt{N_{x}N_{y}N_{z}}.$$

The spectral resolution, represented by the FWHM, can be easily converted into the corresponding $T_{2}^{*}$ relaxation constant using Equation 7—assuming a Lorentzian spectral peak shape. The Δ*B*_0_ considered in Equation 3 leads to deviations from this Lorentzian shape inside the voxel, which add additional Gaussian contributions that finally result in in vivo resonances being Voigt‐shaped. Nevertheless, linewidths can still be well approximated by Equation 7.

(7) $$\Delta f=\frac{1}{\pi T_{2}^{*}}.$$

### 2.3 Quality‐of‐the‐fit parameters

To allow (absolute) metabolite concentrations to be quantified, MRS/MRSI spectra are fit by parametric spectral fitting using e.g., LCmodel.^24^, ^25^ The quality of fit for an unbiased estimator is commonly represented by Cramer‐Rao bounds, which describe the precision of the parameter estimates from the spectra.^26^ Cramer‐Rao lower bounds (CRLBs) describe the lowest variance of the estimated concentration of the metabolites in a given spectrum.^24^ Low CRLBs may not necessarily reflect reliable or accurate results, but they are a reasonable metric for the quality of fit in the absence of a known concentration gold standard.

If the ground truth concentration of metabolites is known, the estimated concentration from the fit can be directly compared. The absolute‐percentage‐error (APE) can be calculated for whole MRSI data sets by Equation 8, where $c_{\mathrm{met}}$ is the vector of concentration values.

(8) $${\mathrm{APE}}_{\mathrm{met}}=\frac{\left|c_{\mathrm{met}}-c_{met,ground\;truth}\right|}{c_{met,ground\;truth}}\times 100.$$

## 3 METHODS

### 3.1 Experimental data

All MRSI simulations were based on acquired data (i.e., Δ*B*_0_ maps, anatomic maps, and fat maps), which were used to simulate FID signals of metabolites and lipids. For this purpose, the same 5 healthy volunteers (age = 24–33 y, 4 male, 1 female) were measured on 4 MR scanners of different *B*_0_ (1.5T Magnetom Aera, 3T Magnetom Prisma, 7T Magnetom, and 9.4T Magnetom; all Siemens Healthineers, Erlangen, Germany), with 4 different coils (1.5T, 20‐channel receive coil array and 3T, 64‐channel receive coil array both with body coils for transmission, both Siemens Healthineers; 7T, a 32‐channel receive coil array combined with a volume coil for transmission, Nova Medical, Wilmington, MA; 9.4T, a 31‐channel receive coil with a 16‐channel parallel transmit coil system).^27^ Internal review board approval and written informed consent were obtained from all volunteers.

The scan protocols were similar for all scanners, but optimized for each respective *B*_0_. Δ *B*_0_ maps were obtained using the same 3D gradient echo sequence with the following parameters: FOV = 220 mm × 192.5 mm, slice thickness = 1.7 mm, matrix size = 128 × 112 × 80, nominal voxel size = 1.7 × 1.7 × 1.7 mm^3^, GRAPPA = 2, TR = 18.0 ms, TE_1_ = 3 ms, and TE_2_ = 6 ms. Only TE_3_ was *B*_0_‐dependent: TE_3,1.5T_ = 14 ms, TE_3,3T_ = 12 ms, TE_3,7T_ = 10 ms, and TE_3,9.4T_ = 9.5 ms to account for faster phase evolution at higher *B*_0_. Multichannel data were combined using the ASPIRE coil combination.^28^ Phase wraps were unwrapped using UMPIRE,^29^ and the Δ*B*_0_ maps were calculated from the difference between phase images.^30^, ^31^ Fat maps were measured only once, on the 3T Prisma, using a turbo spin echo‐based Dixon method^32^ with the same spatial resolution: FOV = 220 mm × 192.5 mm, slice thickness = 1.72 mm, matrix size = 128 × 112 × 80, nominal voxel size = 1.7 × 1.7 × 1.7 mm^3^, TR = 6090 ms, and TE = 14 ms. 3D T_1_‐weighted MPRAGE^33^ or MP2RAGE^34^ were acquired for anatomic reference and to generate brain masks. The T_1_‐weighted MRI data acquired at 1.5T, 7T, and 9.4T were, for each volunteer, co‐registered to the 3T data using affine co‐registration (FLIRT, FSL toolbox).^35^

### 3.2 Simulation of MRSI data

The FID signals of metabolites and lipids were simulated via NMRScopeB^36^ using 1 ms hard pulses with no relaxation. Spectral bandwidths (SBW) and vector size of the simulated signals were *B*_0_‐dependent: ${\text{SBW}}_{1.5\text{T}}=713\text{Hz},{\text{SBW}}_{3\text{T}}=1383\text{Hz},$ ${\text{SBW}}_{7\text{T}}=3333\text{Hz},$ ${\text{SBW}}_{9.4\text{T}}=4488\text{Hz},$ ${\text{vector size}}_{1.5\text{T}}=328,$ ${\text{vector size}}_{3\text{T}}=637,$ ${\text{vector size}}_{7\text{T}}=1536$, and ${\text{vector size}}_{9.4\text{T}}=2068$. The metabolite FIDs contained N‐acetylaspartate (NAA), N‐acetylaspartylglutamate (NAAG), creatine (Cr), phoshocreatine (PCr), phospocholine (PCh), glutamine (Gln), glutamate (Glu), glycerophospocholine (GPC), and myo‐inositol (m‐Ins). Contributions of different metabolites were weighted to achieve similar relative concentrations to in vivo brain spectra. The FIDs of lipids consisted of 5 main resonances, in accordance with Seeger et al.^37^ The effects of apparent *T*_2_ relaxation were simulated by exponential filtering using apparent *T*_2_ constants of the human brain listed in Supporting Information Table S1.^38^, ^39^, ^40^, ^41^, ^42^, ^43^, ^44^, ^45^, ^46^ The apparent *T*_2_ relaxation of lipid components was assumed to be *B*_0_‐independent, as suggested by published values in adipose breast tissue.^47^, ^48^, ^49^, ^50^, ^51^

**Figure 1:**
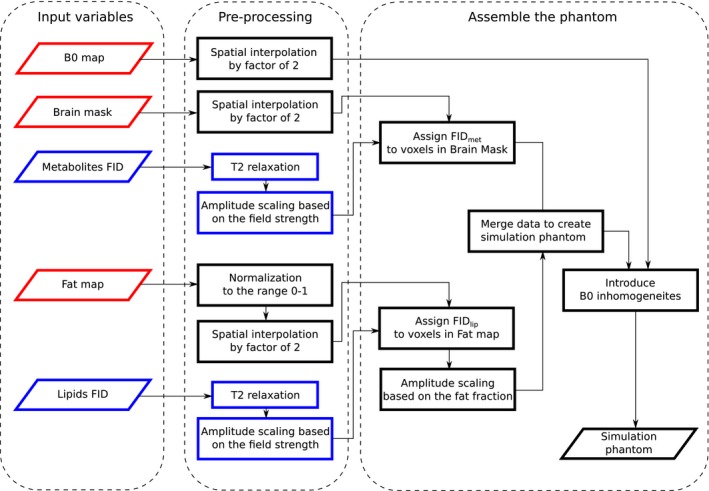
Flowchart of the creation of the simulation phantom from experimentally measured data. The red boxes indicate *B*_0_‐dependent variables and/or steps and the blue boxes indicate volunteer‐independent data

For each volunteer, 4 simulation models (1 for each *B*_0_) were created by performing the same 4 steps in the following order:

1. Δ*B*_0_ maps, fat maps, and brain masks were spatially interpolated to a nominal voxel size of 0.86 × 0.86 × 0.86 mm^3^.
2. The same metabolite FID was assigned to every voxel inside the brain mask.
3. The lipid FID was assigned to each voxel in the fat mask, but the amplitude of the lipid FID was scaled based on the respective fat fraction indicated by the fat map.
4. Δ*B*_0_ obtained from respective experimental Δ*B*_0_ maps were applied via Equation 9, where term ${\text{FID}}_{0}\left(\text{x},\text{y},\text{z},\text{t}\right)$ represents the FID signal of the initial MRSI model at a specific position $\left(\text{x},\text{y},\text{z}\right)$ and term ${\Delta B}_{0}\left(\text{x},\text{y},\text{z}\right)$ represents the *B*_0_ deviation in this voxel. The creation of the simulation phantom is summarized in Figure 1.

(9) $$FID\left(x,y,z,t\right)=FID_{0}\left(x,y,z,t\right)e^{i2\pi \Delta B_{0}\left(x,y,z\right)t}.$$

For each simulation model and volunteer, MRSI acquisitions with different spatial resolutions were simulated in the k‐space‐time‐domain (kx, ky, kz, t) by cutting the inner part of the k‐space to achieve the desired spatial resolutions and adding Gaussian noise. Three different spatial resolutions were simulated, with isotropic voxels of a nominal size: 10 × 10 × 10 mm^3^, 5 × 5 × 5 mm^3^, and 2.5 × 2.5 × 2.5 mm^3^. The SD of the Gaussian noise was SBW‐dependent (i.e., scaled based on Equation 5). Finally, the simulated MRSI data were spatially filtered by a Hamming window and fitted using LCModel.^24^

### 3.3 Evaluation

**Figure 2:**
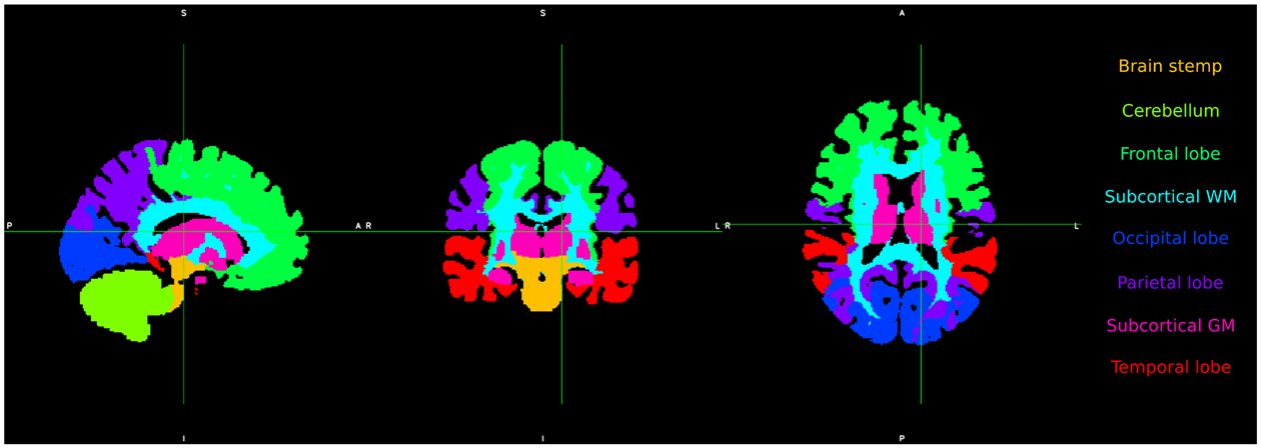
Separation of brain volume into 8 color‐coded regions

For the data evaluation, ~200 regions of interest (ROIs) were obtained from T_1_‐weighted MRI scans using Freesurfer’s cortical and white matter parcellation (APARC).^52^ The ROIs were merged into 8 large regions: frontal lobe, parietal lobe, occipital lobe, temporal lobe, cerebellum, subcortical white matter (WM), subcortical gray matter (GM), and brain stem and then were also co‐registered to 3T image space (Figure 2).

The quality of spectra was characterized in terms of the FWHM and the SNR normalized to voxel volume. FWHM was calculated via a MATLAB script from the LCModel’s fit of NAA, but not directly obtained from the LCmodel output. SNR was computed as the amplitude of the fitted NAA peak divided by the SD of the noise, which was calculated from a metabolite‐free region of the spectra between 5 and 6 ppm. SNR values were then normalized to the voxel volume (nSNR) of the highest spatial resolution by factors derived from Equation 6. The nSNR and FWHM maps of the high and the intermediate‐resolution were resampled to the low resolution to allow pairwise comparison of the results between different spatial resolutions.

CRLBs and APEs were used as quality parameters for the fit and to evaluate quantification accuracy, which inherently contains the effects of lipid leakage through the PSF. Voxels in which the CRLBs of metabolites were higher than 10% were excluded because of low precision. Because the input concentration values in the simulation model were constant across the whole brain, it was expected that the output values would also be constant. The gold standard for estimation of APEs was the median metabolite concentration across those voxels in which CRLBs were below 10%. APEs of metabolites were calculated for every voxel. CRLBs were used to calculate the confidence interval of concentration values (concentration value ± 2*CRLB). Voxels in which the APEs of metabolites were higher than 2 times the CRLB were excluded (low accuracy). Finally, voxels with sufficient quality were counted and converted to volume. The volume after APE thresholding was compared to the volume after CRLB thresholding for both metabolite values and their ratios.

### 3.4 Statistics

The FWHM and nSNR values from all volunteers were merged to create a large data set for each combination of *B*_0_ and spatial resolution. For every *B*_0_, results from different spatial resolutions were compared using the Wilcoxon signed‐rank test. All tests were performed on the data from the whole volume and different anatomical sub‐regions. For the comparison of the 3 spatial resolutions, the resulting *P*‐values were corrected using the Bonferroni correction and the level of significance was assumed at *P* ≤ 0.05.

## 4 RESULTS

**Figure 3:**
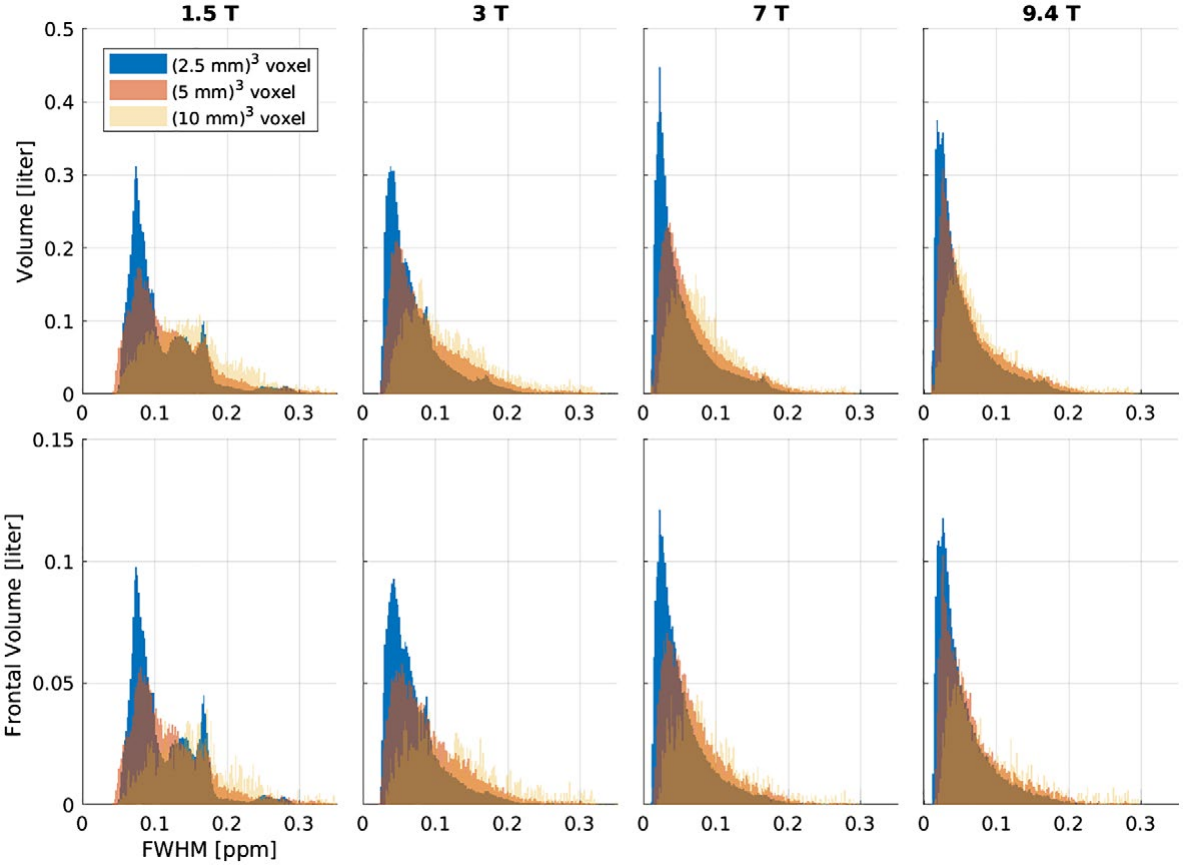
Histogram of FWHM values for all 5 volunteers, normalized to the volume covered. The top row depicts the results from the whole volume and the bottom row shows the results from the frontal lobe, which is most strongly affected by spatial Δ*B*_0_. Each column represents 1 particular *B*_0_

**Figure 4:**
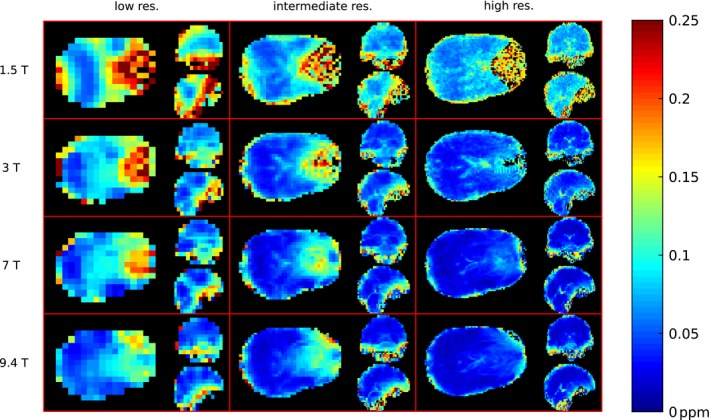
FWHM maps of 1 representative volunteer. Orthogonal slices of the same locations are depicted for 3 different resolutions (rows) and 4 *B*_0_ field strengths (columns). For every subplot the axial slice is depicted on the left side, the sagittal in the top right corner, and coronal in the bottom right corner

The results of the FWHM evaluation are presented in Figure 3 in the form of histograms normalized to the covered volume. With increasing *B*_0_, the histograms of FWHM values shift toward lower FWHM values (in ppm). For example, the medians of the distributions of the FWHM from the intermediate resolution were 0.106, 0.074, 0.055, and 0.050 ppm for 1.5T, 3T, 7T, and 9.4T, respectively. The increase of spatial resolution shifted the FWHM distributions in the histograms to lower values as well. For example at 7T, the median values of FWHM distributions were 0.073, 0.055, and 0.038 ppm for the low, intermediate, and high resolution, respectively. Moreover, the FWHM distributions became narrower, with interquartile ranges (IQR) at 7T of 0.062, 0.058, and 0.045 ppm for low, intermediate, and high resolution, respectively. This effect can also be seen on the FWHM maps in Figure 4, which show that the most pronounced linewidth improvements with increased spatial resolution are achieved in regions of strong Δ*B*_0_, such as the frontal lobe. At 7T, the hotspot of high FWHM values in the frontal lobe (~0.1 ppm) at intermediate resolution was mitigated by increasing spatial resolution similar to the FWHM values found in other sub‐regions (~0.05 ppm). The situation was similar at 3T; the hotspot at intermediate resolution (up to 0.15 ppm) was reduced, although a residuum of the hotspot was present. The median FWHM in different brain regions (after downsampling to the same spatial resolution) and the contribution to this FWHM that originated from Δ*B*_0_ (in %) are summarized in Table 1.

**Table 1:** The results of FWHM analysis

Field strength	Resolution/*P*‐ values	Frontal lobe (%)	Parietal lobe (%)	Occipital lobe (%)	Temporal lobe (%)	Cerebellum (%)	Sub. cortical WM (%)	Sub. cortical GM (%)	Brain stem (%)	Whole brain (%)
1.5T	Low	0.20 (93)	0.18 (92)	0.15 (90)	0.13 (89)	0.14 (90)	0.10 (86)	0.17 (92)	0.14 (90)	0.14 (90)
	Intermediate	0.17 (91)	0.15 (90)	0.10 (86)	0.08 (83)	0.11 (86)	0.08 (81)	0.13 (89)	0.13 (89)	0.10 (86)
	High	0.12 (88)	0.12 (88)	0.08 (83)	0.08 (81)	0.08 (83)	0.07 (81)	0.09 (85)	0.10 (86)	0.08 (83)
	Low vs. inter.	<0.001	<0.001	<0.001	<0.001	<0.001	<0.001	<0.001	<0.001	<0.001
	Low vs. high	<0.001	<0.001	<0.001	<0.001	<0.001	<0.001	<0.001	<0.001	<0.001
	High vs. inter	<0.001	<0.001	<0.001	<0.001	<0.001	0.997	<0.001	<0.001	<0.001
3T	Low	0.16 (94)	0.12 (92)	0.11 (91)	0.09 (90)	0.06 (85)	0.09 (89)	0.13 (93)	0.11 (92)	0.10 (90)
	Intermediate	0.11 (91)	0.10 (90)	0.07 (87)	0.06 (83)	0.05 (82)	0.05 (82)	0.10 (90)	0.09 (90)	0.07 (86)
	High	0.08 (88)	0.07 (86)	0.05 (82)	0.04 (77)	0.05 (79)	0.04 (78)	0.07 (85)	0.07 (86)	0.05 (81)
	Low vs. inter.	<0.001	<0.001	<0.001	<0.001	<0.001	<0.001	<0.001	<0.001	<0.001
	Low vs. high	<0.001	<0.001	<0.001	<0.001	<0.001	<0.001	<0.001	<0.001	<0.001
	High vs. inter	<0.001	<0.001	<0.001	<0.001	<0.001	<0.001	<0.001	<0.001	<0.001
7T	Low	0.12 (95)	0.07 (92)	0.08 (92)	0.07 (91)	0.05 (88)	0.07 (91)	0.10 (94)	0.08 (92)	0.07 (92)
	Intermediate	0.09 (93)	0.07 (92)	0.05 (88)	0.04 (85)	0.05 (86)	0.04 (84)	0.08 (92)	0.07 (91)	0.05 (88)
	High	0.06 (90)	0.05 (87)	0.03 (82)	0.03 (76)	0.03 (78)	0.02 (74)	0.05 (87)	0.05 (88)	0.03 (81)
	Low vs. inter.	<0.001	0.019	<0.001	<0.001	<0.001	<0.001	<0.001	0.062	<0.001
	Low vs. high	<0.001	<0.001	<0.001	<0.001	<0.001	<0.001	<0.001	<0.001	<0.001
	High vs. inter	<0.001	<0.001	<0.001	<0.001	<0.001	<0.001	<0.001	<0.001	<0.001
9.4T	Low	0.12 (94)	0.08 (90)	0.07 (88)	0.06 (87)	0.05 (85)	0.05 (83)	0.10 (92)	0.08 (90)	0.06 (88)
	Intermediate	0.10 (92)	0.07 (89)	0.05 (83)	0.04 (80)	0.04 (81)	0.03 (73)	0.08 (90)	0.08 (90)	0.05 (83)
	High	0.07 (88)	0.05 (85)	0.03 (76)	0.03 (70)	0.03 (73)	0.02 (65)	0.05 (84)	0.06 (86)	0.03 (76)
	Low vs. inter.	<0.001	0.040	<0.001	<0.001	<0.001	<0.001	<0.001	0.916	<0.001
	Low vs. high	<0.001	<0.001	<0.001	<0.001	<0.001	<0.001	<0.001	<0.001	<0.001
	High vs. inter	<0.001	<0.001	<0.001	<0.001	<0.001	<0.001	<0.001	<0.001	<0.001

The FWHM results of the high and intermediate resolution of all *B*_0_ fields were down‐sampled to the low spatial resolution to allow pair‐wise comparison. Median FWHM values [ppm] together with fractions attributed to the Δ*B*_0_ within voxels and *P*‐values from a comparison of different spatial resolutions for brain sub‐regions as well as whole brain are presented.

Median FWHM values decreased with increasing spatial resolution at each *B*_0_ and in most brain regions. Comparing the median values of the FWHM over whole brains, the decrease caused by increasing spatial resolution was the highest at 7T (~2.3‐fold reduction from 0.075 ppm for the low resolution to 0.033 ppm for the high resolution; *P* < 0.001) and was lowest at 1.5T (~1.6‐fold reduction from 0.139 ppm for the low resolution to 0.084 ppm for the high resolution; *P* < 0.001). In a few brain regions, the increase in spatial resolution had no effect on the FWHM. For instance, there were no significant differences at 7T (*P* = 0.06) and 9.4T (*P* = 0.92) in the brain stem region between low and intermediate resolution or at 1.5T in the subcortical WM region between intermediate and high resolution (*P* = 0.997).

**Figure 5:**
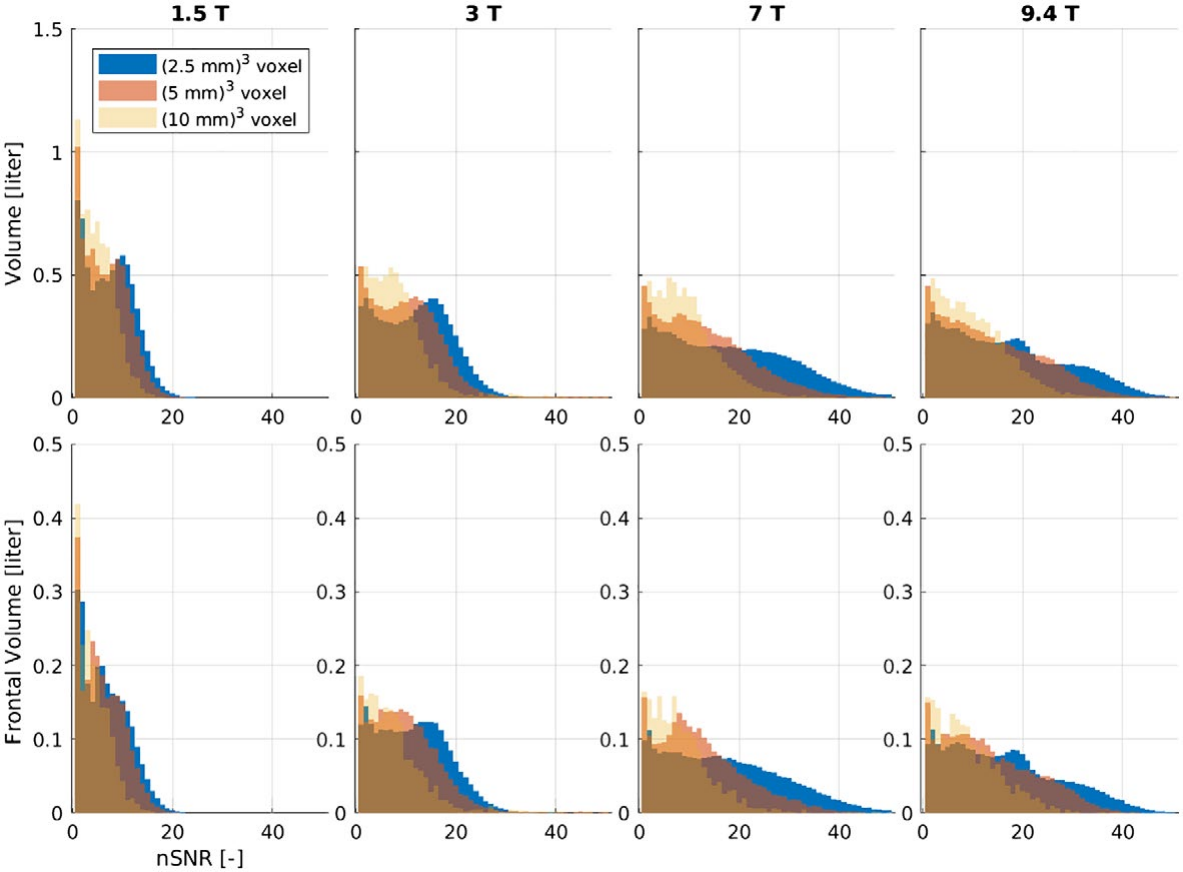
Histograms of nSNR values for all 5 volunteers normalized to the volume covered. The top row depicts the results from the whole volume and the bottom row shows the results from the frontal lobe, which is most strongly affected by spatial Δ*B*_0_. Every column represents 1 particular *B*_0_

**Figure 6:**
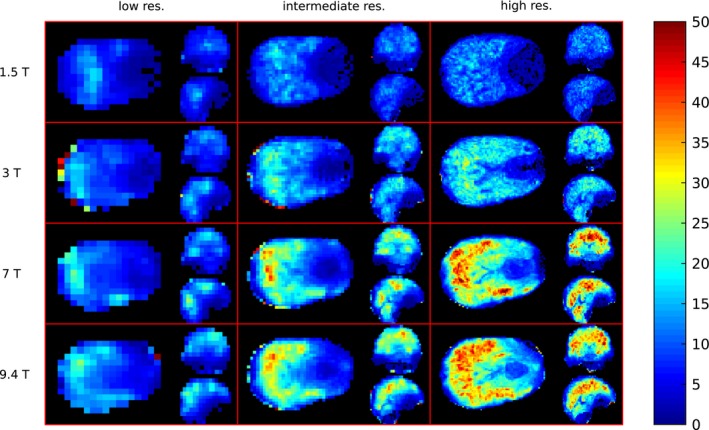
nSNR maps of the same volunteer as depicted in Figure 4. Orthogonal slices of the same locations are depicted for 3 different resolutions (rows) and 4 *B*_0_ field strengths (columns). For every subplot the axial slice is depicted on the left side, the sagittal in the top right corner, and coronal in the bottom right corner

The results of the nSNR are presented in Figure 5 in the form of histograms normalized to the covered volume. For higher *B*_0_, the right tail of the nSNR distributions was shifted toward higher nSNR values. For instance, the third quartiles for the intermediate resolution were 9.26, 14.17, 18.41, and 20.58 for 1.5T, 3T, 7T, and 9.4T, respectively. The nSNR distributions became smaller and broader, meaning that the increase of nSNR was not generalized in the whole volume. This effect was also reflected by an increase of the IQRs (e.g., for the intermediate resolution the IQRs were 6.87, 9.86, 13.07, and 15.33 for 1.5T, 3T, 7T, and 9.4T, respectively). The same was also true for the increase of spatial resolution. The extent of the shift was not consistent among all scanners of different *B*_0_. The highest change was found at 7T. The median values of the nSNR were 7.83, 11.24, and 16.46 for the low, intermediate, and high resolution, while the IQRs were 8.20, 13.07, and 19.45, respectively. Maps of nSNR values are depicted in Figure 6. Median nSNR values are summarized in Table 2, together with the *P*‐values from statistical tests. Almost all *P*‐values were <0.05 except for the comparison of high and intermediate resolution at 1.5T in the frontal lobe (*P* = 0.36).

**Table 2:** The results of nSNR analysis

Field strength	Resolution/*P*‐values	Frontal lobe	Parietal lobe	Occipital lobe	Temporal lobe	Cerebellum	Sub. cortical WM	Sub. cortical GM	Brain stem	Whole brain
1.5T	Low	1.991	2.359	3.367	6.057	5.356	6.686	3.034	3.188	4.461
	Intermediate	3.533	2.540	5.957	8.723	6.800	9.470	6.043	4.055	6.779
	High	3.471	3.242	7.024	9.214	8.958	9.991	6.966	6.148	7.872
	Low vs. inter.	<0.001	<0.001	<0.001	<0.001	<0.001	<0.001	<0.001	<0.001	<0.001
	Low vs. high	<0.001	<0.001	<0.001	<0.001	<0.001	<0.001	<0.001	<0.001	<0.001
	High vs. inter	0.360	<0.001	<0.001	<0.001	<0.001	<0.001	<0.001	<0.001	<0.001
3T	Low	2.437	5.084	6.413	7.884	10.742	9.395	4.725	5.177	7.162
	Intermediate	5.322	6.346	9.607	12.362	12.273	13.514	7.541	5.677	10.192
	High	5.872	8.150	12.437	15.073	15.639	14.452	9.366	8.849	12.820
	Low vs. inter.	<0.001	<0.001	<0.001	<0.001	<0.001	<0.001	<0.001	<0.001	<0.001
	Low vs. high	<0.001	<0.001	<0.001	<0.001	<0.001	<0.001	<0.001	<0.001	<0.001
	High vs. inter	0.006	<0.001	<0.001	<0.001	<0.001	<0.001	<0.001	<0.001	<0.001
7T	Low	2.746	5.820	6.921	9.706	11.842	10.556	5.344	5.436	8.034
	Intermediate	6.203	6.857	11.778	15.724	14.100	17.070	8.932	5.870	12.133
	High	9.259	11.953	18.352	22.193	23.563	25.039	12.422	9.952	18.728
	Low vs. inter.	<0.001	<0.001	<0.001	<0.001	<0.001	<0.001	<0.001	<0.001	<0.001
	Low vs. high	<0.001	<0.001	<0.001	<0.001	<0.001	<0.001	<0.001	<0.001	<0.001
	High vs. inter	<0.001	<0.001	<0.001	<0.001	<0.001	<0.001	<0.001	<0.001	<0.001
9.4T	Low	2.798	4.816	7.607	10.717	11.716	14.633	5.330	4.739	8.787
	Intermediate	6.105	6.202	13.087	17.028	15.351	21.924	8.407	5.282	12.860
	High	7.925	9.377	17.504	21.256	19.224	25.542	11.459	8.873	16.976
	Low vs. inter.	<0.001	<0.001	<0.001	<0.001	<0.001	<0.001	<0.001	<0.001	<0.001
	Low vs. high	<0.001	<0.001	<0.001	<0.001	<0.001	<0.001	<0.001	<0.001	<0.001
	High vs. inter	<0.001	<0.001	<0.001	<0.001	<0.001	<0.001	<0.001	<0.001	<0.001

The nSNR results of the high and intermediate resolution of all *B*_0_ fields were downsampled to the low spatial resolution to allow pairwise comparison. Median of nSNR of brain sub‐regions as well as whole brain and *P*‐values from a comparison of spatial resolutions are presented.

**Figure 7:**
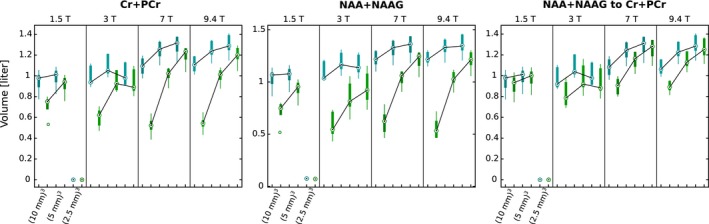
Boxplot for the brain volumes with sufficient data quality for all volunteers. The blue color codes volumes thresholded by CRLBs and the green color codes the volumes thresholded by APE. Data from all 4 field strengths and 3 resolutions are presented. In every subplot, the first pair of boxplots is the result for the lowest resolution, the second is the result for the intermediate resolution, and the third is the result for the highest spatial resolution. Medians of boxplots thresholded by the same parameter are connected. The results on the 1.5T for all 3 cases did not fulfill quality criteria

The volumes in percentage for which the quality‐of‐fit parameter criteria (CRLBs <10% and APE <20%) were fulfilled are presented in Table 3 for 2 metabolites and their ratios. The fraction of brain volumes with sufficient quality increased with increasing spatial resolution in both metrics. The increase was larger in the case of APE. For example, for tNAA at 7T, the fractions were 37%, 63%, and 74% for low, intermediate, and high resolution, respectively, whereas for CRLBs, the fractions were 74%, 80%, and 82% for the same resolutions. The fractions thresholded by CRLBs increased with increasing *B*_0_ field for both metabolites and each spatial resolution by 10–15% when increasing the *B*_0_ field from 1.5T to 9.4T. However, the fractions thresholded by APE increased with increasing *B*_0_ field only for intermediate (+6% by increasing *B*_0_ from 1.5T to 7T and 9.4T) and high resolution (+16% by increasing *B*_0_ from 3T to 7T and 9.4T). In case of low resolution, the decrease was up to 10%. In general, the fractions were lower in case of APE for both metabolites. However, by taking the ratio between tNAA and tCr the fractions thresholded by both metrics were similar especially at 7T and 9.4T for high spatial resolution with ~78%. The difference between volumes thresholded by CRLBs and by APEs for tCr and tNAA increased with increasing *B*_0_ and decreased with increasing spatial resolution (Figure 7).

**Table 3:** Field and resolution dependence of quality parameters

Metabolites	Resolution	CRLBs				APE			
		1.5T (%)	3T (%)	7T (%)	9.4T (%)	1.5T (%)	3T (%)	7T (%)	9.4T (%)
tNAA	Low	63	67	74	75	44	36	37	34
	Intermediate	65	73	80	81	57	52	63	63
	High	4	71	82	83	4	58	74	74
tCr	Low	58	61	67	68	43	36	32	33
	Intermediate	60	67	76	76	56	58	62	61
	High	0	62	79	79	0	57	73	73
tNAA/tCr	Low	57	59	66	66	55	50	55	55
	Intermediate	60	66	75	76	59	60	70	70
	High	0	62	79	79	0	57	77	77

Percentages of the brain volume with sufficient quality thresholded by CRLBs or APE of tNAA, tCr, and its ratio for 3 spatial resolutions at 4 *B*_0_ fields. 100% is equal to the volume of brain fitted by highest spatial resolution. The presented values are the means of the 5 volunteers.

## 5 DISCUSSION

In this study, we successfully modeled MRSI data sets of various spatial resolutions and *B*_0_ field strengths using experimentally obtained data from 5 volunteers to investigate the effects of spatial resolution and *B*_0_ strength on data quality. The main advantage of using simulated data over entirely experimental studies was that the relative metabolite concentrations were known for all data sets and could be used as the gold standard in the evaluation process.

The benefit of increasing spatial resolution to prolong $B_{1}^{+}$ has previously been reported in other areas of MRI^53^; for instance, in both GRE $T_{2}^{*}$‐weighted MRI^54^ and GRE‐based echo planar imaging in functional MRI studies at 1.5T and 3T^55^, ^56^, ^57^ as well as at 7T.^58^

In 3D‐MRSI at 4T, Li et al.^13^ have previously reported a linear decrease in linewidth with the decreasing lateral length of cubic voxels. For in vivo measurements, decreasing the voxel volume from 3.4 cm^3^ to 0.42 cm^3^ yielded a linewidth decrease from 19.6 ± 2.7 Hz to 7.7 ± 1.3 Hz for NAA. A similar decrease was reported by Gruber et al.^12^ at 3T using 3D‐MRSI, where a decrease in voxel volume from 0.75 cm^3^ to 0.094 cm^3^ resulted in a linewidth reduction from 5.3 Hz to 2.9 Hz for the Cr‐CH^3^ resonance. The FOV of both of these studies was localized in the subcortical region. In contrast, a single‐voxel spectroscopy study at 4T and 7T by Tkac et al.^41^ reported that when an optimal *B*_0_‐shim is achieved in the parietal or occipital lobe, linewidth can become independent of voxel volume in the range from 1–8 cm^3^. This may be possible with excellent *B*_0_ shim hardware and/or software in some brain regions, but is not achievable with MRSI.

Our results at 3T are in agreement with the linear decrease of FWHM with voxel volume that was reported for MRSI in subcortical regions,^12^, ^13^ and showed a decrease of the linewidth from 11.0 Hz to 6.8 Hz for a voxel volume reduction from 1 cm^3^ to 0.125 cm^3^. A further decrease of voxel volume to 0.015 cm^3^ improved spectral resolution much less (i.e., 5.5 Hz). This suggests that the ability to decrease linewidth by decreasing voxel volume is limited by the finite variation of *B*_0_ within a voxel.^41^ Tkac et al.^41^ reported that at 7T, the contribution of microscopic susceptibility variations to the FWHM was 5.8 Hz, which is in agreement with our results at 7T. In the high spatial resolution case at 7T, the contribution of Δ*B*_0_ in the subcortical WM region was 5.5 Hz. Increasing the spatial resolution generally decreased the Δ*B*_0_‐dependent contribution to the linewidth. However, even at the highest spatial resolution, Δ*B*_0_ constituted the dominant contribution to linewidth (~70–80%). A large portion of this can be explained by tissue heterogeneity, including bulk susceptibility differences between GM, WM, cerebrospinal fluid, and small vessels and the susceptibility anisotropy (i.e., directionally dependent or tensor nature) of myelin.^59^, ^60^ This contribution cannot be eliminated entirely by *B*_0_ shimming.

Region‐wise evaluation of the data revealed the spatial dependency of the FWHM in terms of absolute values of the FWHM as well as the improvement brought about by increasing spatial resolution. In more *B*_0_‐homogenous regions, such as the subcortical WM, increasing spatial resolution from low to intermediate (FWHM decreased at 7T from 0.07 to 0.04 ppm, at 9.4T from 0.05 to 0.03 ppm) was more beneficial than a further increase to high resolution (FWHM decreased, at 7T from 0.04 to 0.02 ppm, at 9.4T from 0.03 to 0.02 ppm). In contrast, regions with severe Δ*B*_0_, such as the frontal lobe, showed less improvement from increasing spatial resolution from low to intermediate (FWHM decreased at 7T from 0.12 to 0.09 ppm and at 9.4T from 0.12 to 0.10 ppm) than by increasing resolution further from intermediate to high (FWHM decreased at 7T from 0.09 to 0.06 ppm and at 9.4T from 0.10 to 0.07 ppm). This may indicate that very high spatial resolutions are required to truly mitigate intra‐voxel dephasing in the most problematic brain regions.

Because narrower resonance lines lead to higher SNR, the voxel size has a direct impact on SNR. Previous studies reported the decrease of SNR to be less than expected for smaller voxel volumes. Gruber et al.^12^ reported only a 5‐fold SNR decrease for an 8‐fold decrease in voxel volume.

Our results suggest that MRSI becomes more SNR‐efficient per volume with decreasing voxel size—with nSNR being higher in almost all regions for all *B*_0_ fields. However, the increase in nSNR was not homogeneously distributed throughout the brain. The biggest improvements, of more than 100%, were measured in the frontal lobe at 3T, 7T, and 9.4T, with a low‐to‐intermediate increase of spatial resolution. Further increase from intermediate‐to‐high resolution brought up to 50% higher nSNR at 7T. In more *B*_0_‐homogeneous regions, such as the parietal lobe, the situation was different. Between low and intermediate resolution, nSNR increased only by 8% (at 1.5T) to 30% (at 9.4T), but a further increase to high resolution yielded up to 75% improvement (at 7T).

These results suggest that the improvement in FWHM and nSNR by increasing spatial resolution is complex and depends not only on the region of the brain and tissue heterogeneity, but also on the initial and final spatial resolution.

We found a consistent drop in FWHM (on the ppm scale) with increasing *B*_0_ field from 1.5T to 9.4T, in agreement with an experimental study by Otazo et al.^61^ This result was confirmed by a single‐voxel MRS simulation study by Deelchand et al.^8^

In our results, a region‐wise comparison of the FWHM revealed very similar improvement in brain regions with increasing *B*_0_. For the intermediate resolution, increasing *B*_0_ from 1.5T to 3T yielded an ~30% improvement for all regions except the cerebellum, where improvement was up to 49%. Increasing *B*_0_ from 3T to 7T yielded a further improvement of ~25% for almost all regions except the frontal lobe and cerebellum, where it was ~17%. Surprisingly, the increase of *B*_0_ from 7T to 9.4T had a relatively modest impact of <10% on the FWHM in the parietal, occipital, and temporal lobes, as well as cerebellum. The subcortical WM region showed the highest improvement of ~27%. The consistency of the improvement between different brain regions likely arises from brain structure similarities, whereas deviations from this trend are caused by different *B*_0_ shimming hardware and/or software on each MR scanner.

The fraction of the linewidth, which can be attributed to Δ*B*_0_, slightly decreased with increasing *B*_0_, with the biggest decrease found between 7T to 9.4T, where the fraction decreased by up to 9% for the subcortical WM region; however, the fraction in the frontal lobe remained similar (decreased by 2%). These results indicate that, in regions with strong Δ*B*_0_, there is still the potential to improve linewidth.

With increasing *B*_0_, the nSNR also increased. Comparing 1.5T with 3T for intermediate resolution, the nSNR increased by ~34%, which is far below the theoretically possible increase of 100%. Similar results were published in the experimental SVS MRS study by Barker et al.,^45^ who reported a 28% increased SNR when comparing 1.5T versus 3T. For the intermediate spatial resolution at 3T and above, our nSNR values stabilized at ~12. This result is in agreement with those of Deelchand et al., who reported that the SNR‐dependence on *B*_0_ above 4T leveled off.^8^ However, this effect is not consistent across all brain regions, e.g., the subcortical WM, as well as the temporal and occipital lobes exhibited a slight increase of the nSNR with increasing *B*_0_, even up to 9.4T.

The PSF of any imaging technique that requires the Fourier transform is defined as the Fourier transform of its sampling points.^23^ Even if the number of the sampling points increases (with the spatial resolution), the effects of PSF remain defined in units of voxels. Therefore, the increase of spatial resolution decreases the area (or volume) affected by the lipid leakage through the PSF, which translates into an increase of volume with sufficient data quality. Lipid leakage through the PSF deteriorates data quality, but it does not directly transfer into increased CRLBs. The fractions of the volume thresholded by CRLBs were 2‐fold larger compared to fractions thresholded by APE for low resolution at 7T and 9.4T. These effects can be partially recovered by referring to metabolite ratios.

Summarizing all these effects, MRSI benefits several‐fold from higher spatial resolution: in increased spectral resolution, increased SNR per volume, and reduced lipid leakage. This translates into improved quantification accuracy. The increase in precision is in agreement with Deelchand et al.,^8^ who reported that at higher *B*_0_ improved the number of fitted metabolites and lower metabolite CRLBs can be obtained.

### 5.1 Limitations

In agreement with a recent single‐voxel simulation study by Deelchand et al.,^8^ T_1_ relaxation changes with increasing *B*_0_ were considered negligible. Not all *T*_2_ values of metabolites were found in the literature. Therefore, metabolites with similar *T*_2_ were grouped together and missing values were extrapolated. In practice, these assumptions should not significantly alter our results since additional line‐broadening because of *B*_0_‐ and metabolite‐dependent *T*_2_ contribute only by 0.9 Hz to 3.2 Hz (based on the *B*_0_ for NAA), whereas the effect of Δ*B*_0_ on line‐broadening is in the range of 8–25 Hz. For extracranial lipids, the *T*_2_ values of breast adipose tissue were used, which were available for all field strengths.^47^, ^48^, ^49^, ^50^, ^51^ In any case, our simulations were performed for an FID‐MRSI sequence, which does not have an echo time. Hence, there is no *T*_2_‐related signal decay.

For simplicity, our simulation model assumed spatial phase encoding in all 3 directions. In fact, several much more time‐efficient MRSI encoding strategies exist.^62^, ^63^, ^64^ Our MRSI data were simulated without including B_1_ inhomogeneities, which potentially could have a significant effect on the SNR at higher *B*_0_. We decided to do this because different coil geometries, the number of receive channels, and substantially different transmit coils (i.e., body coils, local transmit coils, or parallel transmit coils) make a fair comparison impossible. In practice, experimental studies will be strongly affected by the choice of coils, as observed previously,^61^ but results reflect an undesirable mix of *B*_0_‐ and coil‐efficiency dependence. Our MRSI data were simulated assuming excellent water suppression. In practice, insufficient water suppression could impair accurate spectral fitting, but simulating this in a more realistic manner requires many assumptions about parameters including water T_1_, B_1_^+^ inhomogeneities, and choice of water suppression technique, which would only distract from the investigation of the *B*_0_ and spatial resolution dependence. This simplification is consistent with a recent single‐voxel simulation study by Deelchand et al.^8^ The SNR was assumed to increase linearly with *B*_0_, which was also measured experimentally by Otazo et al. for birdcage coils, but not multi‐channel coils, where they observed less than linear increase.^61^ In contrast to this, Pohmann et al.^65^ reported SNR to scale supralinearly with *B*_0_ (~*B*_0_^1.65^) for multi‐channel coils, which would yield even higher improvement. In our study, we did not report the absolute SNR values, mainly because we were predominantly interested in the effect of the increasing resolution at different *B*_0_ fields and in that case, the normalization to voxel volume is required. Additionally, the amount of signal compared to noise is inherently covered by CLRBs and APE.

The quantification precision associated with the fitting process was assessed by CRLBs. This is common in the field of MRS/MRSI, although it is not a good measure of the quantification quality. Fortunately, our simulation model also allowed us to estimate quantification accuracy by comparing the confidence interval of estimated metabolite concentrations against the APE. The absolute values of metabolite concentrations (in mmol) were not used as the gold standard. Instead, in our simulation model—in which a constant concentration across the whole volume was introduced at the beginning— the same constant concentration was assumed as a gold standard to quantify the APE.

## 6 CONCLUSIONS

Our study suggests that moving to high spatial resolution, whole‐brain MRSI will improve spectral quality and associated quantification accuracy, which will enable the study of neurochemical changes in the diseased and healthy brain in more detail. Because improvements in data quality were more pronounced at higher *B*_0_ and in more inhomogeneous regions, high resolution and further improved *B*_0_ shimming hardware can be expected to jointly overcome the challenges associated with ultra‐high field and thereby enable clinically robust whole‐brain MRSI at 7T and higher.

## Supporting information


**TABLE S1 **The *B*_0_ dependent apparent *T*_2_ relaxation time constants of metabolites and lipid components used for simulations

## References

[1] Kirov II , Liu S , Tal A , et al. Proton MR spectroscopy of lesion evolution in multiple sclerosis: Steady‐state metabolism and its relationship to conventional imaging. Hum Brain Mapp. 2017;38:4047–4063. 28523763 10.1002/hbm.23647 PMC5510951

[2] Öz G , Alger JR , Barker PB , et al. Clinical proton MR spectroscopy in central nervous system disorders. Radiology. 2014;270:658–679. 24568703 10.1148/radiol.13130531 PMC4263653

[3] Andronesi OC , Loebel F , Bogner W , et al. Treatment response assessment in IDH‐mutant glioma patients by noninvasive 3D Functional spectroscopic mapping of 2‐hydroxyglutarate. Clin Cancer Res. 2016;22:1632–1641. 26534967 10.1158/1078-0432.CCR-15-0656 PMC4818725

[4] Zanigni S , Testa C , Calandra‐Buonaura G , et al. The contribution of cerebellar proton magnetic resonance spectroscopy in the differential diagnosis among parkinsonian syndromes. Parkinsonism Relat Disord. 2015;21:929–937. 26077167 10.1016/j.parkreldis.2015.05.025

[5] Voevodskaya O , Sundgren PC , Strandberg O , et al. Myo‐inositol changes precede amyloid pathology and relate to APOE genotype in Alzheimer disease. Neurology. 2016;86:1754–1761. 27164711 10.1212/WNL.0000000000002672 PMC4862247

[6] Fleischer V , Kolb R , Groppa S , Zipp F , Klose U , Gröger A . Metabolic patterns in chronic multiple sclerosis lesions and normal‐appearing white matter: intraindividual comparison by using 2D MR spectroscopic imaging. Radiology. 2016;281:536–543. 27243371 10.1148/radiol.2016151654

[7] Juchem C , de Graaf RA . B0 magnetic field homogeneity and shimming for in vivo magnetic resonance spectroscopy. Anal Biochem. 2017;529:17–29. 27293215 10.1016/j.ab.2016.06.003 PMC5148734

[8] Deelchand DK , Iltis I , Henry P‐G . Improved quantification precision of human brain short echo‐time (1) H magnetic resonance spectroscopy at high magnetic field: a simulation study. Magn Reson Med. 2014;72:20–25. 23900976 10.1002/mrm.24892 PMC3907456

[9] Stockmann JP , Wald LL . In vivo B0 field shimming methods for MRI at 7 T. Neuroimage. 2018;168:71–87. 28602943 10.1016/j.neuroimage.2017.06.013 PMC5760477

[10] Stockmann JP , Witzel T , Keil B , et al. A 32‐channel combined RF and B0 shim array for 3T brain imaging. Magn Reson Med. 2016;75:441–451. 25689977 10.1002/mrm.25587 PMC4771493

[11] Ebel A , Maudsley AA . Improved spectral quality for 3D MR spectroscopic imaging using a high spatial resolution acquisition strategy. Magn Reson Imaging. 2003;21:113–120. 12670597 10.1016/s0730-725x(02)00645-8

[12] Gruber S , Mlynárik V , Moser E . High‐resolution 3D proton spectroscopic imaging of the human brain at 3 T: SNR issues and application for anatomy‐matched voxel sizes. Magn Reson Med. 2003;49:299–306. 12541250 10.1002/mrm.10377

[13] Li B , Regal J , Gonen O . SNR versus resolution in 3D1H MRS of the human brain at high magnetic fields. Magn Reson Med. 2001;46:1049–1053. 11746567 10.1002/mrm.1297

[14] Hingerl L , Strasser B , Moser P , et al. Towards full‐brain {FID}‐{MRSI} at 7T with 3D concentric circle readout trajectories In Proceedings of the Joint Annual Meeting of ISMRM‐ESMRMB, Paris, France, 2018. Abstract 0618.

[15] Nassirpour S , Chang P , Henning A . whole brain high resolution metabolite mapping using 1H FID {MRSI} with slice‐wise B0 shim updating at 9.4T In Proceedings of the Joint Annual Meeting of ISMRM‐ESMRMB, Paris, France, 2018. Abstract 0619.

[16] Heckova E , Strasser B , Hangel GJ , et al. 7 T magnetic resonance spectroscopic imaging in multiple sclerosis. Invest Radiol. 2019;54:247–254. 30433892 10.1097/RLI.0000000000000531 PMC7612616

[17] Hangel G , Jain S , Hečková E , et al. Patch‐based super‐resolution of 7 T {MRSI} of glioma: initial results In Proceedings of the Joint Annual Meeting ISMRM‐ESMRMB, Paris, France, 2018. Abstract 0451.

[18] Parra NA , Maudsley AA , Gupta RK , et al. Volumetric spectroscopic imaging of glioblastoma multiforme radiation treatment volumes. Int J Radiat Oncol Biol Phys. 2014;90:376–384. 25066215 10.1016/j.ijrobp.2014.03.049 PMC4346247

[19] Govind V , Gold S , Kaliannan K , et al. Whole‐brain proton MR spectroscopic imaging of mild‐to‐moderate traumatic brain injury and correlation with neuropsychological deficits. J Neurotrauma. 2010;27:483–496. 20201668 10.1089/neu.2009.1159 PMC2867627

[20] Levin BE , Katzen HL , Maudsley A , et al. Whole‐brain proton MR spectroscopic imaging in Parkinson’s disease. J Neuroimaging. 2014;24:39–44. 23228009 10.1111/j.1552-6569.2012.00733.x PMC4593470

[21] Donadieu M , Le Fur Y , Lecocq A , et al. Metabolic voxel‐based analysis of the complete human brain using fast 3D‐MRSI: Proof of concept in multiple sclerosis. J Magn Reson Imaging. 2016;44:411–419. 26756662 10.1002/jmri.25139 PMC4940345

[22] Michaeli S , Garwood M , Zhu X‐H , et al. ProtonT2 relaxation study of water, N‐acetylaspartate, and creatine in human brain using Hahn and Carr‐Purcell spin echoes at 4T and 7T. Magn Reson Med. 2002;47:629–633. 11948722 10.1002/mrm.10135

[23] De Graaf RA . Wiley InterScience (Online service). In vivo NMR spectroscopy: principles and techniques. Hoboken: Wiley‐Interscience. 2007;592:p.

[24] Provencher SW . Estimation of metabolite concentrations from localized in vivo proton NMR spectra. Magn Reson Med. 1993;30:672–679. 8139448 10.1002/mrm.1910300604

[25] Vanhamme L , Van Den Boogaart A , Van HS . improved method for accurate and efficient quantification of MRS data with use of prior knowledge. J Magn Reson. 1997;129:35–43. 9405214 10.1006/jmre.1997.1244

[26] Cavassila S , Deval S , Huegen C , Van Ormondt D , Graveron‐Demilly D . Cramé r‐Rao bound expressions for parametric estimation of overlapping peaks: influence of prior knowledge. J Magn Reson. 2000;143:311–320. 10729257 10.1006/jmre.1999.2002

[27] Shajan G , Kozlov M , Hoffmann J , Turner R , Scheffler K , Pohmann R . A 16‐channel dual‐row transmit array in combination with a 31‐element receive array for human brain imaging. Magn Reson Med. 2014;71:870–879. 23483645 10.1002/mrm.24726

[28] Eckstein K , Dymerska B , Bachrata B , et al. computationally efficient combination of multi‐channel phase data from multi‐echo acquisitions (ASPIRE). Magn Reson Med. 2018;79:2996–3006. 29034511 10.1002/mrm.26963

[29] Robinson S , Schödl H , Trattnig S . A method for unwrapping highly wrapped multi‐echo phase images at very high field: UMPIRE. Magn Reson Med. 2014;72:80–92. 23901001 10.1002/mrm.24897 PMC4062430

[30] Robinson S , Jovicich J . *B* _0_ mapping with multi‐channel RF coils at high field. Magn Reson Med. 2011;66:976–988. 21608027 10.1002/mrm.22879

[31] Jezzard P , Balaban RS . Correction for geometric distortion in echo planar images from B0 field variations. Magn Reson Med. 1995;34:65–73. 7674900 10.1002/mrm.1910340111

[32] Dixon WT . Simple proton spectroscopic imaging. Radiology. 1984;153:189–194. 6089263 10.1148/radiology.153.1.6089263

[33] Mugler JP , Brookeman JR . Three‐dimensional magnetization‐prepared rapid gradient‐echo imaging (3D MP RAGE). Magn Reson Med. 1990;15:152–157. 2374495 10.1002/mrm.1910150117

[34] Marques JP , Gruetter R . New developments and applications of the MP2RAGE sequence–focusing the contrast and high spatial resolution R1 mapping. PLoS One. 2013;8:e69294. 23874936 10.1371/journal.pone.0069294 PMC3712929

[35] Jenkinson M , Bannister P , Brady M , Smith S . Improved optimization for the robust and accurate linear registration and motion correction of brain images. Neuroimage. 2002;17:825–841. 12377157 10.1016/s1053-8119(02)91132-8

[36] Starčuk Z , Starčuková J , Štrbák O , Graveron‐Demilly D . Simulation of coupled‐spin systems in the steady‐state free‐precession acquisition mode for fast magnetic resonance (MR) spectroscopic imaging. Meas Sci Technol. 2009;20:104033.

[37] Seeger U , Klose U , Mader I , Grodd W , Nägele T . Parameterized evaluation of macromolecules and lipids in proton MR spectroscopy of brain diseases. Magn Reson Med. 2003;49:19–28. 12509816 10.1002/mrm.10332

[38] Scheidegger M , Hock A , Fuchs A , Henning A . T2 relaxation times of 18 brain metabolites determined in 83 healthy volunteers in vivo In Proceedings of the 22nd Annual Meeting of ISMRM, Milan, Italy, 2014. Abstract 2947.

[39] Marjańska M , Auerbach EJ , Valabrègue R , Van de Moortele P‐F , Adriany G , Garwood M . Localized 1H NMR spectroscopy in different regions of human brain in vivo at 7 T: T2 relaxation times and concentrations of cerebral metabolites. NMR Biomed. 2012;25:332–339. 21796710 10.1002/nbm.1754 PMC3357544

[40] Choi C , Coupland NJ , Bhardwaj PP , et al. T2 measurement and quantification of glutamate in human brain in vivo. Magn Reson Med. 2006;56:971–977. 17029225 10.1002/mrm.21055

[41] Tkáč I , Andersen P , Adriany G , Merkle H , Uǧurbil K , Gruetter R . In vivo ^1^ H NMR spectroscopy of the human brain at 7 T. Magn Reson Med. 2001;46:451–456. 11550235 10.1002/mrm.1213

[42] Deelchand DK , Moortele P‐F , Adriany G , et al. In vivo 1H NMR spectroscopy of the human brain at 9.4 T: initial results. J Magn Reson. 2010;206:74–80. 20598925 10.1016/j.jmr.2010.06.006 PMC2940249

[43] Träber F , Block W , Lamerichs R , Gieseke J , Schild HH . ^1^H metabolite relaxation times at 3.0 tesla: measurements of T1 and T2 values in normal brain and determination of regional differences in transverse relaxation. J Magn Reson Imaging. 2004;19:537–545. 15112302 10.1002/jmri.20053

[44] Li Y , Xu D , Ozturk‐Isik E , et al. T1 and T2 metabolite relaxation times in normal brain at 3T and 7T. J Mol Imaging Dynam. 2013;2:1–5.

[45] Barker PB , Hearshen DO , Boska MD . Single‐voxel proton MRS of the human brain at 1.5T and 3.0T. Magn Reson Med. 2001;45:765–769. 11323802 10.1002/mrm.1104

[46] Mlynárik V , Gruber S , Moser E . Proton T (1) and T (2) relaxation times of human brain metabolites at 3 Tesla. NMR Biomed. 2001;14:325–331. 11477653 10.1002/nbm.713

[47] Rakow R , Daniel B , Sawyer‐Glover A , Glover G . T1 and T2 measurements of breast tissue at 0.5 T, 1.5 T and 3.0 T In Proceedings of the 11th Annual Meeting of ISMRM, Toronto, Canada, 2003. Abstract 0167.

[48] Dean KI , Majurin ML , Komu M . Relaxation times of normal breast tissues. Acta Radiol. 1994;35:258–261. 8192964

[49] Dimitrov IE , Douglas D , Ren J , et al. In vivo determination of human breast fat composition by 1 H magnetic resonance spectroscopy at 7 T. Magn Reson Med. 2012;67:20–26. 21656551 10.1002/mrm.22993 PMC3245342

[50] Edden R , Smith SA , Barker PB . Longitudinal and multi‐echo transverse relaxation times of normal breast tissue at 3 Tesla. J Magn Reson Imaging. 2010;32:982–987. 20882630 10.1002/jmri.22306 PMC3154635

[51] Rakow‐Penner R , Daniel B , Yu H , Sawyer‐Glover A , Glover GH . Relaxation times of breast tissue at 1.5T and 3T measured using IDEAL. J Magn Reson Imaging. 2006;23:87–91. 16315211 10.1002/jmri.20469

[52] Desikan RS , Ségonne F , Fischl B , et al. An automated labeling system for subdividing the human cerebral cortex on MRI scans into gyral based regions of interest. Neuroimage. 2006;31:968–980. 16530430 10.1016/j.neuroimage.2006.01.021

[53] Hezel F , Thalhammer C , Waiczies S , Schulz‐Menger J , Niendorf T . High spatial resolution and temporally resolved *T* _2_* mapping of normal human myocardium at 7.0 Tesla: an ultrahigh field magnetic resonance feasibility study. PLoS One. 2012;7:e52324. 23251708 10.1371/journal.pone.0052324 PMC3522647

[54] Tang MY , Chen TW , Zhang XM , Huang XH . GRE T2∗‐weighted MRI: principles and clinical applications. Biomed Res Int. 2014;2014:312142. 24987676 10.1155/2014/312142 PMC4009216

[55] Chen NK , Dickey CC , Yoo SS , Guttmann CR , Panych LP . Selection of voxel size and slice orientation for fMRI in the presence of susceptibility field gradients: application to imaging of the amygdala. Neuroimage. 2003;19:817–825. 12880810 10.1016/s1053-8119(03)00091-0

[56] Robinson S , Windischberger C , Rauscher A , Moser E . Optimized 3 T EPI of the amygdalae. Neuroimage. 2004;22:203–210. 15110010 10.1016/j.neuroimage.2003.12.048

[57] Robinson SD , Pripfl J , Bauer H , Moser E . The impact of EPI voxel size on SNR and BOLD sensitivity in the anterior medio‐temporal lobe: a comparative group study of deactivation of the Default Mode. MAGMA. 2008;21:279–290. 18661163 10.1007/s10334-008-0128-0

[58] Speck O , Stadler J , Zaitsev M . High resolution single‐shot EPI at 7T. MAGMA. 2008;21:73–86. 17973132 10.1007/s10334-007-0087-x

[59] Lee J , Shmueli K , Fukunaga M , et al. Sensitivity of MRI resonance frequency to the orientation of brain tissue microstructure. Proc Natl Acad Sci U S A. 2010;107:5130–5135. 20202922 10.1073/pnas.0910222107 PMC2841900

[60] Wharton S , Bowtell R . Fiber orientation‐dependent white matter contrast in gradient echo MRI. Proc Natl Acad Sci U S A. 2012;109:18559–18564. 23091011 10.1073/pnas.1211075109 PMC3494918

[61] Otazo R , Mueller B , Ugurbil K , Wald L , Posse S . Signal‐to‐noise ratio and spectral linewidth improvements between 1.5 and 7 Tesla in proton echo‐planar spectroscopic imaging. Magn Reson Med. 2006;56:1200–1210. 17094090 10.1002/mrm.21067

[62] Mansfield P . Spatial mapping of the chemical shift in NMR. Magn Reson Med. 1984;1:370–386. 6571566 10.1002/mrm.1910010308

[63] Furuyama JK , Wilson NE , Thomas MA . Spectroscopic imaging using concentrically circular echo‐planar trajectories in vivo. Magn Reson Med. 2012;67:1515–1522. 22006586 10.1002/mrm.23184

[64] Adalsteinsson E , Irarrazabal P , Topp S , Meyer C , Macovski A , Spielman DM . Volumetric spectroscopic imaging with spiral‐basedk‐space trajectories. Magn Reson Med. 1998;39:889–898. 9621912 10.1002/mrm.1910390606

[65] Pohmann R , Speck O , Scheffler K . Signal‐to‐noise ratio and MR tissue parameters in human brain imaging at 3, 7, and 9.4 tesla using current receive coil arrays. Magn Reson Med. 2016;75:801–809. 25820458 10.1002/mrm.25677

