# Supplementary material for: The influence of spatial resolution on the spectral quality and quantification accuracy of whole‐brain MRSI at 1.5T, 3T, 7T, and 9.4T
Source: Magn Reson Med. 2019 Apr 1;82(2):551–65. doi: 10.1002/mrm.27746 (PMC6563461; doi:10.1002/mrm.27746)
Supplement: Supplementary file 1 — TABLE S1 The B 0 dependent apparent T 2 relaxation time constants of metabolites and lipid components used for simulations [file MRM-82-551-s001.docx]

**Supporting Information**

Table S1: The B0-dependent apparent T2 relaxation time constants of metabolites and lipid components used for simulations

| Metabolite | Field Strength | | | |
| --- | --- | --- | --- | --- |
|  | 1.5T | 3 T | 7 T | 9.4 T |
| NAA,NAAG,mIns | 350 ms | 262 ms | 170 ms | 100 ms |
| Cr,Pcr | 213 ms | 150 ms | 121 ms | 70 ms |
| Cho,PCho,GPC | 320 ms | 270 ms | 131 ms | 70 ms |
| Glu,Gln | 228 ms | 260 ms | 105 ms | 60 ms |
| Lipids (1-5) | 50 ms | 50 ms | 50 ms | 50 ms |
